# Influence of Connectivity, Wild Prey and Disturbance on Occupancy of Tigers in the Human-Dominated Western Terai Arc Landscape

**DOI:** 10.1371/journal.pone.0040105

**Published:** 2012-07-05

**Authors:** Abishek Harihar, Bivash Pandav

**Affiliations:** 1 School of Anthropology and Conservation, Durrell Institute of Conservation and Ecology, University of Kent, Canterbury, Kent, United Kingdom; 2 Wildlife Institute of India, Dehradun, India; Australian Wildlife Conservancy, Australia

## Abstract

Occupying only 7% of their historical range and confined to forested habitats interspersed in a matrix of human dominated landscapes, tigers (*Panthera tigris*) typify the problems faced by most large carnivores worldwide. With heads of governments of tiger range countries pledging to reverse the extinction process and setting a goal of doubling wild tiger numbers by 2022, achieving this target would require identifying existing breeding cores, potential breeding habitats and opportunities for dispersal. The Terai Arc Landscape (TAL) represents one region which has recently witnessed recovery of tiger populations following conservation efforts. In this study, we develop a spatially explicit tiger occupancy model with survey data from 2009–10 based on *a priori* knowledge of tiger biology and specific issues plaguing the western TAL (6,979 km^2^), which occurs in two disjunct units (Tiger Habitat Blocks; THBs). Although the overall occupancy of tigers was 0.588 (SE 0.071), our results clearly indicate that loss in functionality of a regional corridor has resulted in tigers now occupying 17.58% of the available habitat in THB I in comparison to 88.5% in THB II. The current patterns of occupancy were best explained by models incorporating the interactive effect of habitat blocks (AIC *w* = 0.883) on wild prey availability (AIC *w* = 0.742) and anthropogenic disturbances (AIC *w* = 0.143). Our analysis has helped identify areas of high tiger occupancy both within and outside existing protected areas, which highlights the need for a unified control of the landscape under a single conservation unit with the primary focus of managing tigers and associated wildlife. Finally, in the light of global conservation targets and recent legislations in India, our study assumes significance as we identify opportunities to secure (e.g. THB II) and increase (e.g. THB I) tiger populations in the landscape.

## Introduction

Fragmentation and the loss of connectivity between suitable habitats have led to range wide population declines among many mammalian species [1]. In particular, the interactive effects of an expanding human population and innate biological traits of mammalian carnivores make them vulnerable to extinction [2]–[5]. Studies have demonstrated that effects of fragmentation are most severely realized by larger carnivores, which thereby are not being able to persist in human dominated landscapes with weak or no linkages to suitable habitat tracts [6]. Given their critical role in ecosystems, large carnivores often serve as umbrella species, garnering support for conservation, and also serve as effective focal species to assess the impact of anthropogenic disturbances across large landscapes [7]. Tigers (*Panthera tigris* Linnaeus, 1758) typify the problems faced by most large carnivores worldwide. Occurring across many parts of Asia around 200 years ago, they now occupy only 7% of their original range owing to habitat loss, prey depletion and direct persecution [8], [9]. Most of these populations are now confined to forested habitats interspersed in a matrix of human dominated landscape. While studies have delineated and prioritized landscapes as the best options for securing tiger meta-populations for long-term conservation [8], ensuring population persistence requires that landscapes remain permeable to tiger movement, and source sites are secured within them [10], [11].

With heads of governments of the 13 tiger range countries pledging to reverse the extinction process and setting a goal of doubling wild tiger numbers by 2022 [12], optimistic recovery scenarios suggest that priority tiger conservation landscapes represent sufficient habitat to support such targets [11]. One such landscape with the potential to support far greater numbers is the Terai-*bhabhar* habitats that were once contiguous along the base of the Himalayas in India and Nepal. Historically these regions supported dense populations of tigers and their prey that probably formed a single interbreeding population [13]. However, more recently studies have recognized five subpopulations, based on land-cover data and ground surveys, with poor or no connectivity between them owing to unnatural breaks in habitat [14], [15]. Although tigers can disperse over large distances from their natal areas to establish territories, they are reluctant to cross more than a few kilometers of unsuitable land cover [16]. While recommendations provided by Johnsingh et al. [14] and Wikramanayake et al. [15] for managing regional dispersal corridors and forests outside reserve boundaries have helped recover tiger populations in the recent past [17], [18], the lack of timely action in securing vital corridors such as the one across the Gola river has further severed habitat connectivity in the Terai Arc Landscape (TAL) [11]. Although the alluvial savannah/grassland habitats still hold some of the largest concentrations of tigers across their range, most populations are confined to protected areas covering 25% of the land area in this linear landscape. Recent local extinction events have reiterated the fact that small reserves alone are inadequate and landscape scale approaches that ensure the expansion of the number of breeders beyond existing cores is required to maintain viable populations [19].

**Figure 1:**
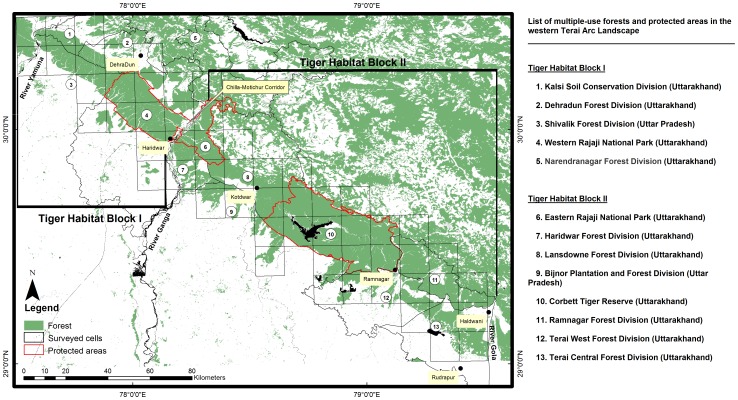
Potential tiger habitat in the western Terai Arc Landscape. Framed within 57 grid cells (166 km^2^) spanning the area between river Yamuna and river Gola are the two Tiger Habitat Blocks (THB’s). Also indicated are the administrative units highlighting the protected areas of Rajaji National Park (RNP) and Corbett Tiger Reserve (CTR), the Chilla-Motichur corridor along the river Ganga and the major towns/cities in the area.

The western end of this region, forming the range limit of tigers, spans from river Yamuna in the west to river Gola in the east and occurs in two disjunct units, termed as Tiger Habitat Blocks (THBs; [14]), owing to poor or no habitat connectivity across the Chilla-Motichur corridor (Figure 1). Covering 2,925 km^2^, THB I comprises of 4 multiple-use forest divisions (FD) and the western sector of Rajaji National Park [RNP] (570 km^2^). The remaining section of the landscape spanning 4,054 km^2^ (THB II), comprises of 2 protected areas (eastern sector of RNP: 250 km^2^ and Corbett Tiger Reserve [CTR]: 1288 km^2^) and 6 FDs (Figure 1). Our previous studies suggest that a small isolated population of tigers occurs in THB I as opposed to relatively high density populations occurring in THB II (Table 1 & Text S1).

**Table 1:** Summary of population sizes estimated from camera-trapping studies conducted in the western TAL.

	Sites	Number of individualsidentified	Estimatedpopulation size	Trap area (sq. km)	Density (SE)per 100 km^2^
THB I					
	Western RNP^a^	2	2 (0.3)	266	0.4 (0.1)
THB II					
	Eastern RNP^a^	7	9 (0.9)	133	5.6 (1.6)
	Lansdowne FD^a^	9	10 (1.6)	101	6.1 (2.3)
	Corbett National Park^b^	101	109 (5.4)	611	16.2 (1.6)
	Ramnagar FD^b^	26	27 (1.5)	177	13.8 (2.7)

a See Text S1 for details.

b Jhala et al. [24].

In this study, we developed a spatially-explicit tiger occupancy model using a survey design with correlated spatial replication within large geographic grid cells following Hines et al. [20]. To understand the factors influencing occupancy of tigers across the 6,979 km^2^ of potential tiger habitat in the western TAL, we confront predictions with survey data based on *a priori* knowledge of tiger biology and specific issues plaguing the landscape [14], [21], [22].

### Predictions

Being obligate carnivores, occupancy of tigers should be positively influenced by availability of wild prey species [22]. Although domestic livestock (chiefly buffalo *Bubalus bubalis* Linnaeus, 1758 and cattle *Bos taurus* Bojanus, 1827) often forms the diet of tigers, studies specific to this landscape have indicated that they depress natural prey densities and potentially render the habitat unsuitable for tigers [17]. In addition, a vast body of literature supports that human activities negatively influence tiger occupancy, confining most breeding cores or high tiger use areas to well managed protected areas [14], [21]–[23]. In this study we draw upon these generalities and use field data indexing prey availability and chronic disturbances from the western TAL to specifically test the following predictions.

#### *Prediction 1: Lack of breeding and/or connectivity to a source would result in lower occupancy rates of tigers in THB I*

Surveys conducted in 2002–03 by Johnsingh et al. [14] documented that tigers occurred more widely in THB I. However, more recent studies [21] have shown that a few isolated individuals occur in western RNP. Although THB I could form an ideal dispersal ground for tigers from the newly established source in eastern RNP [17] or CTR (a high density source [24]), we anticipate that the current lack of connectivity would result in a lower estimated proportion area occupied by tigers in contrast to THB II. This assumes conservation significance as populations at their range limits are more susceptible to local extinctions.

#### *Prediction 2: Occupancy of tigers would be governed by the availability of wild prey and more specifically principal prey*

In the western TAL sambar (*Rusa unicolor* Kerr, 1792), nilgai (*Boselaphus tragocamelus* Pallas, 1766), chital (*Axis axis* Erxleben, 1777), wild pig (*Sus scrofa* Linnaeus, 1758), hog deer (*Hyelaphus porcinus* Zimmermann, 1780), goral (*Naemorhedus goral* Hamilton Smith, 1827), barking deer (*Muntiacus muntjak* Zimmermann, 1780), porcupine (*Hystrix indica* Kerr, 1792), langur (*Semnopithecus hector* Pocock, 1928) and hare (*Lepus nigricollis* Cuvier, 1823) form the potential wild prey species of tigers. Although wild prey assemblages are relatively intact within protected areas, these species occur at lower densities in multiple use forests. Tiger densities are known to be a function of prey biomass and density [25], [26]. Therefore, following Karanth et al. [22], we included the frequency occurrence of wild prey as a covariate and predict that occupancy responses to wild prey availability would be positive. In general, studies from parts of this landscape reveal that sambar and chital are the most numerically dominant prey species in the prey assemblages (68%) and also occur more commonly in the diet of tigers (70–78%) [17], [21], [27]. Therefore, we consider them as principal prey in further analyses and test the predictive ability of this covariate in explaining the observed patterns in tiger occupancy.

#### *Prediction 3: Despite the negative influence of anthropogenic disturbances on tiger occupancy, individuals in THB II would be forced to occupy sub-optimal habitats*

The western TAL is characterized by resource pressures such as livestock grazing, illegal hunting of wild prey and collection of fuel wood and non-timber forest produce (e.g. *bhabar* grass *Eulaliopsis binata*) [14]. With 13% (i.e. 400 km^2^ of western RNP) of the potential tiger habitat currently free of anthropogenic disturbances (i.e. inviolate), THB I, supports a small population of tigers (Table 1). In contrast, THB II, with 16.5% inviolate habitat (i.e. 520 km^2^ of CTR and 150 km^2^ of eastern RNP) harbors a high density of tigers (Table 1). Given that tigers in THB II occur at high densities even outside protected areas, we predict that individuals in THB II would be forced to occupy sub-optimal habitats (areas of low prey availability and high anthropogenic disturbances) although human activities would negatively influence tiger occupancy.

#### *Prediction 4: Field collected covariates would better explain the occupancy patterns than remotely sensed surrogates*

Recent studies assessing tiger distributions in the TAL use surrogate measures of habitat and disturbance from remotely sensed data [28]. However, we predict that the relative contribution (Akaike weight; *w_i_*) of field data indexing prey availability and chronic disturbances would serve as better predictors of occupancy than remotely sensed habitat surrogates.

## Materials and Methods

### Ethics Statement

Permissions to conduct field research were obtained from the state forest departments of Uttarakhand and Uttar Pradesh under the provisions of Section-12 and Section-28 of the Wildlife (Protection) Act, 1972 and the Guidelines for Scientific Research in Protected Areas, Ministry of Environment and Forests, Government of India.

### Study Area

Our study area almost entirely falls in the Shivalik and *bhabar* tract characterized by rugged hills ranging from 600 m to 1200 m with a low water table and streams disappearing into permeable sediments. The overall land cover matrix consists of natural forests interspersed with agricultural and forestry crops. The natural vegetation consists of both moist as well as dry deciduous forests, with the north facing slopes dominated by Sal (*Shorea robusta*) and the south facing slopes covered by mixed forests comprising of tree species such as *Terminalia alata, Anogeissus latifolia, Lagerstroemia parviflora, Holoptelia integrifolia, Ehretia laevis* and *Aegle marmelos* [29]. Extensive grasslands of *Saccharum* spp occur in relatively undisturbed valleys and shorter grasslands comprising of *Imperata cylindrica*, *Chrysopogon aciculatus* and *Eragrostis* spp. occur in intensely grazed areas. Further details regarding the vegetation associations and land cover matrix of the study area can be found in Johnsingh et al. [14].

The study area supports ca. 6.9 million people [30] and is characterized by multiple resource pressures on the habitat [14]. In addition, *Gujjars*, a group of nomadic pastoralists, inhabit many areas of this landscape. Although some are still nomadic, coming down to the Shivalik and *bhabar* tract during the winter and returning to high elevation pastures in the Himalayas during summer, most reside permanently within these forests. Permits are issued to families living within multiple-use forests to cut grass and lop branches off trees for leaves to provide fodder to their livestock holdings. Such resource extractions are not permitted in the protected areas of Corbett National Park and RNP. Primarily consisting of buffaloes, their livestock holdings are large and so are their requirements for fodder. Furthermore, rapid urban growth in cities such as Dehra Dun, Haridwar, Kotdwar, Ramnagar, Haldwani and Rudrapur are severing already tenuous corridors [14], [21].

### Survey Design

To assess the occupancy of tigers in the western TAL, we adopted a survey design with correlated spatial replication being used in large scale occupancy surveys for tigers [20], [22], [31], [32]. Our sampling units consisted of a grid of geographical cells overlaid on the land-cover matrix of the study area (Figure 1). As our goal was to estimate the proportion of area occupied rather than the intensity of habitat use by tigers, we chose a cell size (166 km^2^) larger than the maximum home range size of ∼60 km^2^ documented in similar habitats [33]. For ease of conducting the field surveys, the boundaries of the cells coincided with grid lines on the 1∶25,000 topographic maps of Survey of India.

Typically, occupancy modelling requires moderate to large sample sizes to achieve precise estimates [34], [35]. In a previous application of the Hines et al. [20] model, inferences applied to a landscape matrix of 38,350 km^2^ framed by 205 survey cells [22]. However, in this study, our objective was to map tiger distribution across 6,979 km^2^ of potential habitat framed by 57 cells (Figure 1). Therefore, to test for sample size adequacy simulated data and numeric-analytic approximations were generated using the program GENPRES [36].

Following the survey design outlined by Hines et al. [20], we fixed the maximum survey distance as 40 km if the cell entirely comprised tiger habitat based on the per cent forest cover. This distance was proportionately reduced depending on the extent of habitat. Thereby, effort ranged from 4 km of walk in cells with ∼10% forest cover to 40 km of walk in cells with 100% forest cover. The survey routes were planned in advance to ensure adequate spatial coverage of cells by randomly choosing one location in the cell through which surveyors would pass.

### Field Data Collection

Our surveys were conducted over 5 months to minimize the likelihood of changes in occupancy during sampling. In addition, each cell was surveyed within <12–48 hrs to meet the assumption of closure. From November 2009 to March 2010, we surveyed a total 1166 km along dry sandy streambeds, forest roads and trails recording tracks, dung deposits and territorial markings of target species as well as signs of disturbance. *A priori* knowledge of abundance and distribution suggested that tigers were less abundant in THB I and possibly in the non-protected regions of THB II as well [14], [21], [27]. Therefore, routes within cells were surveyed randomly to ensure that regions with differing abundances were sampled under similar survey conditions. Based on earlier work [27], which demonstrates that parts of the study area experiences considerable inter-annual variation in ungulate and tiger densities, we chose to sample only during the dry winter months. Given that the average precipitation during this period is ∼48 mm, we feel that variations in sign detection rates induced by rainfall would be minimum.

All surveys were conducted by a team of four experienced surveyors, each with more than 5 years’ experience in conducting sign surveys in this region. Though studies [37] have noted that occupancy estimation based on signs are subject to the decay rates of signs, in this study we assume that sign availability was approximately equal across the study area. As tigers typically walk ∼1–20 km/day along travel routes to hunt or mark them intensively [33], [38], [39], search along such trails increases detection probabilities beyond that expected from random sampling. During the surveys we searched for signs of tigers along survey routes at a rate of 10–15 km/day. To avoid any potential misidentification of signs (distinguishing tigers from leopards), tracks and scrapes were identified using a combination of size, shape, depth and gait, while scats were identified based on their size and the presence of secondary evidences at the site. We spent adequate time to verify the signs and only recorded signs that were unambiguously identified. The data were recorded along segments of 250 m as either detected (1) or not-detected (0).

As we predicted that occupancy of tigers would be positively influenced by wild ungulate prey and negatively influenced by disturbance variables, we also collected field based site-specific covariate data during our surveys with the assumption that detectability of covariates influencing tiger presence remained constant across the study area. Tracks of wild prey (sambar, nilgai, chital, hog deer, wild pig, barking deer, goral, langur, porcupine and hare), domestic livestock (buffalo, cattle, goats/sheep) and signs of human activities (lopping, wood cutting) were recorded along the 250 m segments as ‘1’ or ‘0’. These were then summarized, variable-wise, to signify the proportion of 1 km replicates containing the sign. For the purpose of analyses, cell specific proportions of each wild prey species were summed to represent the overall wild prey index (WildP; Figure S1). Similarly, a cell specific index of principal prey (PrincipP; Figure S2) was computed as the summed proportions of signs of sambar and chital. Finally, a comprehensive disturbance variable (Dist; Figure S3) was computed as the summed proportions of signs of domestic livestock and human activities. While we assumed that this spatial scale these metrics were reliable covariates influencing tiger occurrence in our models, we believe that variations associated with these values did not significant influence parameter estimates. In addition to these ground based variables, we also estimated the proportional habitat (Hab) per cell by super-imposing our sampling grid over the land-cover matrix [14] using ArcGIS 9.3. Although survey effort was planned based on the proportional habitat (Hab) in each cell, the realized effort (in km) varied. Therefore, to model detection probability, we considered the effect of effort in km (E). Replicates were categorized as one in three substrates (S) that favoured detection of sign (Sandy streambeds, forest roads or trails) and given that we expected all parameters of interest in our occupancy analysis to vary by block, a categorical covariate (B) representing cell membership to THB I and II was coded as ‘1’ and ‘0’, respectively.

### Data Analysis

We constructed detection histories for each cell by aggregating signs along 250 m segments at 1 km to form ‘replicates’ and imported both survey and site-specific covariates into program PRESENCE 3.1 [40]. We standardized continuous covariates such as effort, WildP, PrincipP and Dist using z-transformations and treated categorical covariates (block and substrate) as dummy variables with values of 0 or 1, while proportional habitat per cell was incorporated as a covariate without transformation [41]. To avoid multicollinearity, we only selected variables with tolerance levels greater than 0.1, as recommended by Hair et al. [42]. We used a first order Markov process model [20] to estimate occupancy (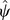) of tigers in the western TAL. Under this formulation segment-level occupancy (parameterised by 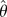 and 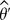) and detection probability (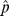 *_t_*) conditional on segment-level occupancy helped explicitly decompose the detection process. For few cells (n = 4), since we had to sequentially combine disjointed trail segments to build detection histories, we chose the analytical variant of Hines et al. model [20] that assumes surveys can begin on any randomly chosen replicate.

In our analysis we used a two-step approach to model parameters of interest. We first began by modelling covariates on detection probability, where the parameter was either assumed constant or allowed to vary with individual or additively combined covariates. Given that variations in abundance could influence sign detection rates [43], we incorporated the effect of blocks, in addition to effort and substrate type, to minimize un-modelled sources of heterogeneity in detection probability. For each model of 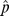 *_t_*, we held 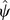 in a general model [44]. We then compared candidate models using Akaike’s Information Criterion (AIC; [45]). In the second step we modelled the influence of covariates on occupancy while incorporating covariates included in detection probability models that contained >90% of Akaike weight (*w_i_*). As we wished to test four ecological/management predictions, we constructed nine models incorporating the relevant covariates (WildP, PrincipP, Dist and Hab) on 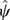 modelled to vary individually or as an interactive effect by block. For all models, segment-level occupancy parameters (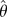 and 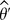) were modelled as varying by block as *a priori* knowledge of abundance and distribution of tiger suggested that tigers were less abundant in THB I as compared to THB II. We computed the model-averaged estimates of cell specific 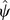 by considering all the plausible alternative occupancy models. To estimate the overall tiger habitat occupancy (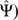 within the western TAL, we weighed the cell-specific occupancy estimates by potential tiger habitat in the cell. The computation of variance followed Karanth et al. [22].

## Results

Our simulation results (Text S2) indicated that bias in occupancy for the final models would be trivial (% bias<|4.5%|) even if the number of primary sampled sites were reduced to 18, thereby indicating that our estimates derived from a total of 57 cells are relatively unbiased.

During our surveys (1166 km walk of trails), we detected tiger signs on 611 one km segments in 32 of the 57 surveyed cells, resulting in a naïve occupancy estimate of 0.56. We recorded 204 individual track sets and collected 89 scats. In all our analysis, segment-level occupancy parameters were modelled on block. As we had assumed that surveys could begin on any randomly chosen replicate, we estimated the probability of presence on the first replicate [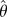
_0_(SE[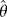 *_0_*])] as 0.394 (0.03) in THB I and 0.794 (0.06) in THB II. However, the probability of presence on replicate, given absence on previous replicate [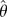
(SE[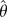])] and the probability of presence on replicate, given presence on previous replicate [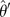
(SE[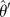])] indicated that in THB I, spatial dependence was weak [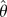 = 0.341 (0.21) and 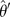 = 0.479 (0.15)]. In contrast, 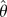 and 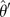 were estimated to be 0.271 (0.05) and 0.929 (0.06) respectively in THB II, indicating strong between-segment dependence.

### Detection Probability

**Figure 2:**
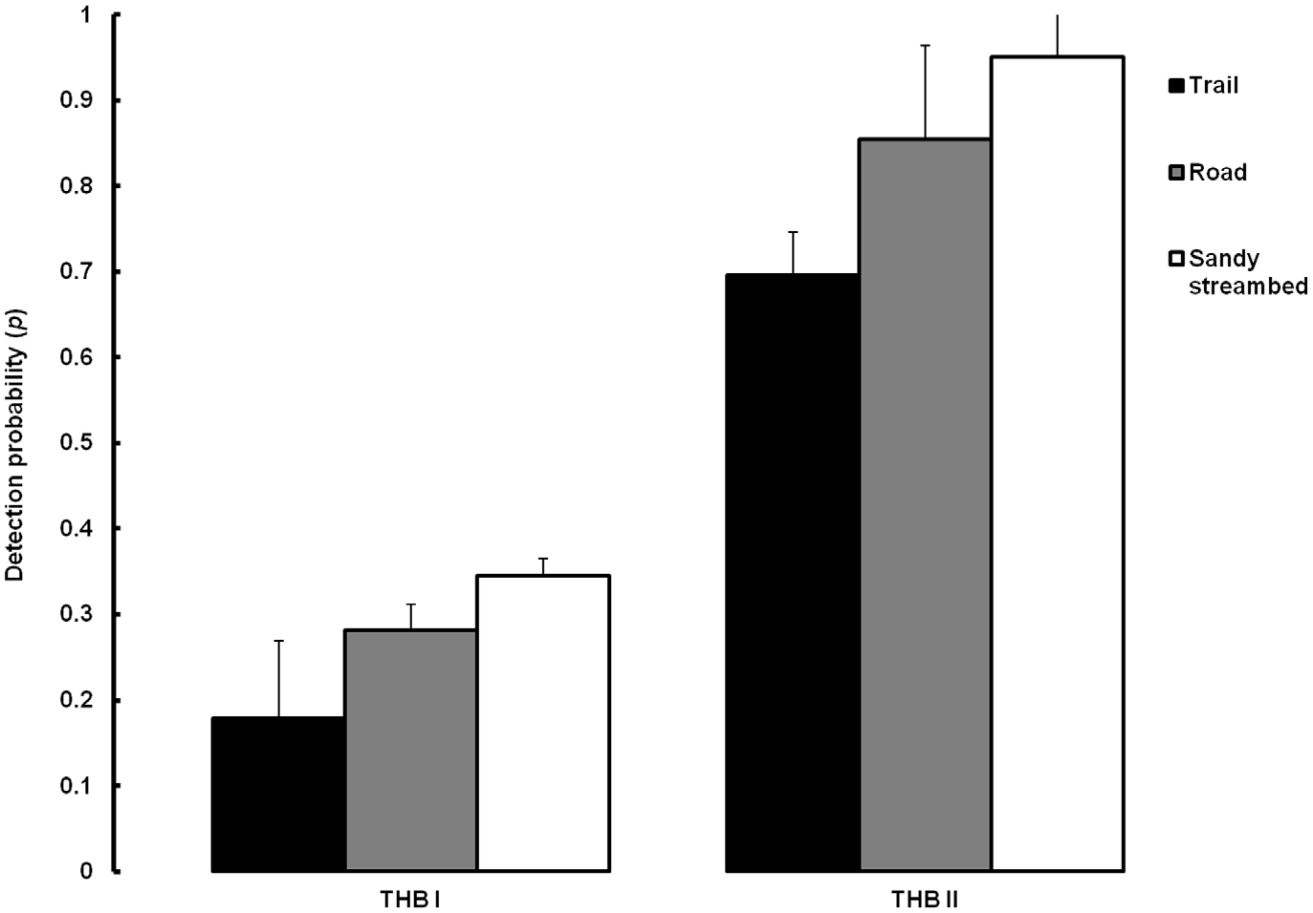
Probability of detecting tiger signs in THB I and THB II as a function of substrate type. Error bars represent one standard error.

Two of our models incorporating block and substrate type had greater support (*w* = 0.901) compared to all 8 plausible alternative models constructed to assess the effect of block, effort and substrate on detection probability (Table 2). As we had hypothesized, the difference in abundance of tigers between habitat-blocks strongly influenced detection probability (*w* = 0.901). The mean segment-level probability of detecting tiger signs [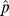 *_t_* (SE[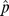 *_t_*])] in THB I was 0.386 (0.08) while in THB II it was estimated to be 0.947 (0.02). As expected, substrate type greatly influenced the detectability of signs (*w* = 0.831; Figure 2). Sandy streambeds, which were the dominant substrate type in 82% of survey segments, had the highest 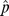 in both habitat blocks (0.345 (0.02); THB I and 0.951 (0.05); THB II). The detection probability along forest roads (15% of surveys) was 0.282 (0.03) in THB I and 0.854 (0.11) in THB II, while along forest trails (3% of surveys), 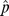 was estimated to be the lowest at 0.179 (0.09) in THB I and 0.696 (0.05) in THB II. Our models indicated that effort had little effect on the detection probability (*w* = 0.001). Therefore, we used the additive model incorporating the effect of block and substrate on detection probability [*p* (B + Substrate)] to examine habitat occupancy (Table 3).

**Table 2:** Effect of covariates^a^ on detection probability (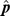).

Model^b^	ΔAIC	AIC weight	Number of parameters	Deviance (-2 Log-likelihood)
*p* (B + Substrate)	0	0.7786	12	837.5
*p* (B)	3.7	0.1224	10	845.2
*p* (Substrate)	5.41	0.0521	11	844.91
*p* (.)	5.65	0.0462	9	849.15
*p* (Effort)	14.92	0.0004	10	856.42
*p* (B + Effort)	16.92	0.0002	11	856.42
*p* (Substrate + Effort)	18.92	0.0001	12	856.42
*p* (B + Substrate + Effort)	20.92	0	13	856.42

Note: Model rankings are based on Akaike’s Information Criterion (AIC).

a Covariates used to model detection probability were Block (B), Substrate (sandy streambeds, roads and trails) and Effort. ‘.’ denotes that was held constant instead of being allowed to vary as a function of any covariate.

b In all models the probability of occupancy (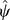) was modelled on ‘B × Hab’ and segment level occupancy parameters (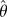 and 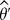) were modelled on ‘B’ (Block). ‘+’ denotes covariates were modelled additively.

**Table 3:** Effect of covariates^a^ on occupancy (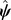).

Model^b^	ΔAIC	AIC weight	Number of parameters	Deviance (-2 Log-likelihood)
*ψ* (B × WildP)	0	0.654	12	837.02
*ψ* (B × Dist)	3.05	0.142	12	840.07
*ψ* (WildP)	4.03	0.087	10	845.05
*ψ* (B × Hab)	4.18	0.081	12	841.2
*ψ* (B)	6.32	0.027	10	847.34
*ψ* (B × PrincipP)	9.81	0.004	12	846.82
*ψ* (Hab)	12	0.001	10	853.02
*ψ* (Dist)	14.69	0.0004	10	855.71
*ψ* (PrincipP)	18.97	0	10	859.99

Note: Model rankings are based on Akaike’s Information Criterion (AIC).

a Covariates used to model detection probability were Block (B), Wild prey index (WildP), Principal prey index (PrincipP), Disturbance (Dist) and proportional habitat per cell (Hab).

b In all models the probability of detection (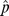) was modelled as ‘B + Substrate’ based on model selection results presented in Table 1. Segment-level occupancy parameters (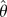 and 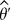) were modelled on ‘B’ (Block). ‘×’ denotes covariates were modelled as an interaction.

### Occupancy Patterns

**Figure 3:**
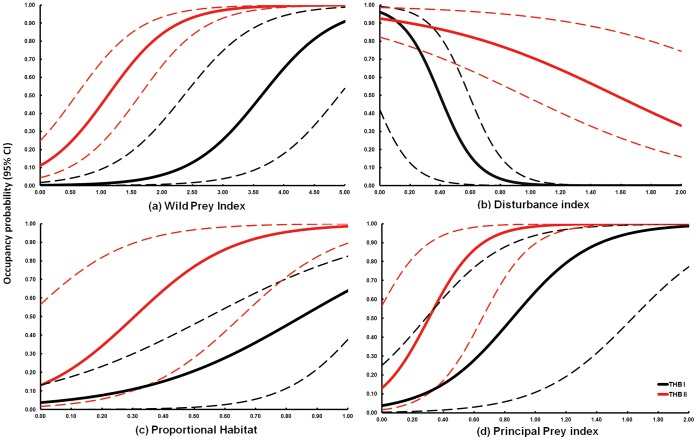
Relationship between occupancy probability (y-axis) and explanatory variables across THB I and THB II. (a) wild prey index, (b) disturbance index, (c) proportional habitat and (d) principal prey index. Dashed lines represent 95% confidence intervals.

**Figure 4:**
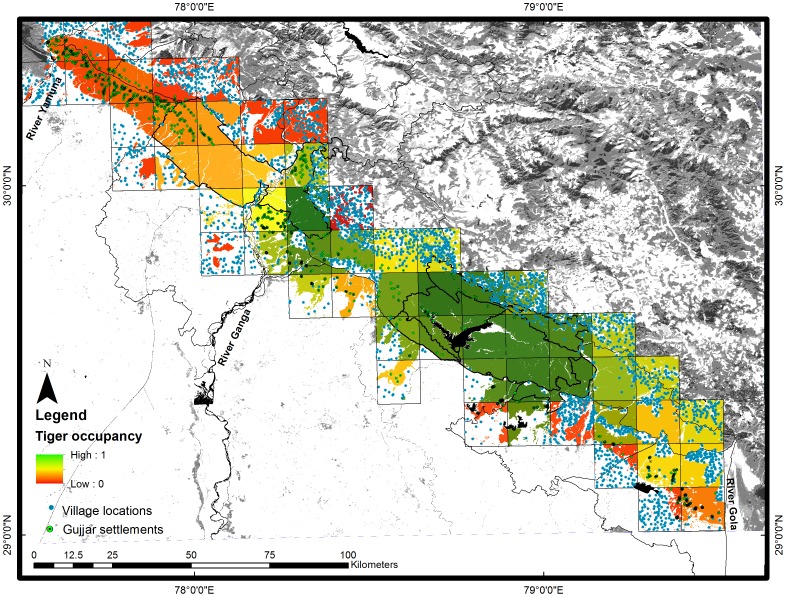
Occupancy of tigers in the western Terai Arc Landscape. Model averaged probability of cell specific occupancy for tigers in relation to human settlements in the western Terai Arc Landscape, India, 2009–10.

The total fraction of area occupied by tigers (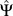
(SE[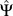])) in the western TAL was 0.588 (0.071), resulting in an area of 4,109 km^2^ (SE = 492 km^2^) of the 6,979 km^2^ of potential habitat. As hypothesized, tiger distribution was influenced by the break in connectivity across the Chilla-Motichur corridor, characterized in our analysis by the high support received for model incorporating the effect of block on 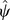 (*w* = 0.911; Table 3). In THB I, we estimated that only 17.85% (522 km^2^; SE = 90 km^2^) of the 2,925 km^2^ of potential habitat was occupied by tigers. In contrast, 88.5% (3,587 km^2^; SE = 237 km^2^) of the 4,054 km^2^ of available habitat was occupied in THB II. Models with only one of the covariates (WildP, PrincipP, Dist and Hab) on cell-specific occupancy performed poorly (*w* = 0.089) compared to those modelled as an interactive effect on habitat blocks (*w* = 0.883), indicating that the effect of covariates on occupancy differed between THBs. In general, models incorporating field based covariates such as wild prey (*w* = 0.742; Table 3) and anthropogenic disturbances (*w* = 0.143; Table 3) received greater support than the remotely sensed surrogate ‘proportional habitat’ (*w* = 0.083; Table 3) with the exception of principal prey (*w* = 0.005; Table 3). In addition, *β* coefficient estimates from the nine models revealed that WildP, Hab and PrincipP positively influenced the presence of tiger, while Dist was a negative influence on occupancy (Figure 3), providing support to our predictions. However, to account for model selection uncertainty, we estimated the cell specific 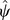 by applying standard model averaging procedures and mapped the geographical variations across the landscape (Figure 4).

## Discussion

Our results demonstrate that tigers now occupy 17.6% of the available habitat in THB I in comparison to 88.5% in THB II within the western TAL. And the current patterns of occupancy were best explained by models incorporating the interactive effect of habitat blocks on wild prey and anthropogenic disturbances.

Estimating occupancy, whilst accounting for detection probability using spatially replicated surveys, has recently gained popularity to assess the status and distribution of tigers [22], [31], [32]. In our particular case, 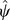 (0.588±0.071) was similar to the naive estimate (0.56) reflecting a small probability of false-negatives [44], thereby highlighting the importance of a good field survey design that accounted for variations in detection rates across the two habitat blocks and three substrate types (sandy streambeds, forest roads and trails). As the *bhabar* habitat provides suitable substrates (esp. sandy streambeds) to aid detect track sets, which are the most numerous signs, we predominantly recorded pug marks (204 sets of tracks versus 89 scat deposits) in contrast to surveys conducted in south-western India where a greater proportion of tiger signs detected were scat deposits [22]. In addition, abundance-induced heterogeneity influenced the estimates of all parameters (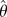, 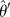 and 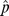) associated with decomposing the probability of detecting tiger presence. Taken together, these results indicate that variations in abundance and substrates, both within and across landscapes, influence inferences on occupancy and studies not accounting for imperfect detections (e.g. [24], [28], [46]), both at the field design and analytical stages, could estimate parameters of interest with substantial bias.

As predicted, models incorporating block (B) as an influence on tiger occupancy in the western TAL received great support despite uncertainties in model selection (Table 3). While patterns of occupancy at the level of the landscape are a response to complex interactions between multiple factors [22], [31], [32], we interpret this result as evidence for loss in connectivity between multiple source populations in THB II to potential habitat in THB I. While traditional analyses [28] have failed to identify population level responses to the loss in connectivity, we have been able to identify the same by testing a relatively small set of candidate models based on spatially explicit and ecologically meaningful predictions.

The occupancy of tigers, as hypothesized, was positively influenced by the availability of wild prey species. Our previous studies have shown that well protected regions of the western TAL support higher wild prey densities in comparison to many sites across the tigers range [17], [21], [26], [27]. Given that the lack of well stratified data on prey availability from outside protected areas has been recognized as a major impediment in extrapolating data to show conditions for tigers outside of reserves [47], the relative abundance index (WildP and PrincipP) developed here presents a comprehensive assessment of wild prey availability across multiple forest management regimes in the western TAL. In particular, our analysis reveals that occupancy varied differentially with wild prey occurrence in THB I and II, reflecting differences in sub-population sizes. Contrary to one of our predictions, we found that sambar and chital (PrincipP) contributed less to the observed patterns in occupancy, although they are numerically dominant and preferred prey within protected areas [17], [21], [27]. We believe that this is reflective of the fact that (a) tigers, although occurring in well protected regions of THB I, currently exist at population densities well below carrying capacities and (b) in THB II, tigers occur at high population densities outside existing protected areas where wild prey densities are potentially depressed owing to livestock mediated competition and other anthropogenic influences.

In support of our prediction, the influence of anthropogenic disturbances on occupancy was negative. In THB I the few remaining tigers actively select for optimal habitats indexed by high wild prey occurrence and low disturbances. However, given high population densities, individuals in THB II were forced to include sub-optimal habitats within their territories. This has led to tigers often coming in conflict with the interests of humans and also led to several incidents of retaliatory killing [48].

A major difference between our approach and a recent study conducted in the TAL [28] is our use of intensive field data indexing wild prey availability and anthropogenic disturbances. As an example, Kanagaraj et al. [28] identify “good habitat” quality across multiple-use forests of THB I by classifying habitats using a combination of two predictive models (“Natural” and “Disturbance”) developed with remotely sensed surrogates. In contrast, our study and prior field surveys [14] suggest that these areas (Kalsi Soil Conservation Division and Dehradun Forest Division), despite possessing dense forest cover support low wild prey numbers which render these habitats sub-optimal for tigers, reaffirming the significance of using field data pertaining to prey species occurrence to model habitat suitability.

### Conservation Implications

Results of this study support earlier findings that available habitat in THB I could serve as ideal dispersal grounds and could potentially support a minimum of 30 individuals [17], [21]. However, the lack of connectivity to source populations east of river Ganges prevents colonization. Despite specific recommendations provided earlier by Johnsingh et al. [14] and Harihar et al. [21] to restore and maintain functionality of this regional corridor, interventions to restore this corridor have begun only recently following the directives of the Honourable Supreme Court of India. Apart from the lack of connectivity to source populations, Johnsingh et al. [14] identified the presence of forest settlements, stealing of kills by *bhabar* grass collectors and poaching of prey as the major threats to this population. However, since 2005 settlements from most parts of western RNP (400 km^2^) have been voluntarily relocated and the subsequent strengthening of protection in these parts has resulted in the cessation of *bhabar* grass collection. In addition, studies by Harihar et al. [21] documenting high prey densities (110 ungulates/km^2^) within western RNP indicate that protection, at least within the park, is effective. Yet the tiger population has not recovered. In comparison to surveys conducted by Johnsingh et al. [14], we confirm a decline in tiger occurrence in THB I within a span of 7 years, further supporting the contention that populations at their range limits are more susceptible to local extinctions and highlighting the fact that the lack of institutional accountability and consequently delayed conservation action could lead to extinction of isolated populations [49]. While a viable strategy to maintain demographic and genetic connectivity across the sub-populations should involve restoring habitat in the corridor region, given the existence of a small and non-viable population of tigers in THB I [21], government and conservation agencies should immediately translocate individuals from a nearby source (e.g. CTR) to prevent the otherwise imminent local extinction.

Since the last assessment of tiger occurrence across the TAL [14], studies have documented recovery in the tiger population in eastern RNP for the following reasons: (a) minimization of anthropogenic pressures which included the voluntary resettlement of *Gujjars* that led to disturbance free habitats safe for tiger to breed in, and (b) connectivity of eastern RNP with CTR through the Rajaji-Corbett corridor (Lansdowne Forest Division) that led to the immigration and subsequent colonization of individuals [17], [27]. This has, therefore, resulted in the establishment of a new globally important source site (eastern RNP; 250 km^2^) within THB II in addition to CTR (1288 km^2^), [10]. Although both protected areas receive substantial financial support from both the state government as well as federally sponsored schemes such as ‘Project Elephant’ and ‘Project Tiger’, these areas require the establishment of standardized patrol based law enforcement monitoring (LEM) to strengthen frontline law enforcement capacity and effectiveness. Since these areas are adequately staffed, equipped and financed, LEM could serve as a tool to provide managers with the information they need to make strategic decisions as direct killing of tigers and their prey are the most immediate concerns in securing tiger populations range wide [50].Our analysis has also helped identify, rapidly and systematically, areas of high tiger occupancy outside existing protected area networks. This represents considerable challenges to land area management as tigers occur in multiple-use forests which are primarily managed under forestry operations and are inhabited and used by pastoralist *Gujjars* and local communities for grazing livestock, collecting fodder and fuel wood. In particular our results highlight that parts of Lansdowne, Bijnor, Ramnagar and Terai west FDs, adjoining CTR show high tiger occupancy similar to the known source sites (RNP and CTR) and also support breeding tigers. However, since management of these habitats is not aligned with conserving wildlife and administrations receive inadequate financial and infrastructural support for training, equipping and deploying staff towards law enforcement. Consequently, protection against poaching is minimal and tigers are particularly susceptible to direct killings despite these regions acting as crucial extensions to the tiger population of CTR. Studies also indicate that these areas experience high conflict, with a reported 645 cases of livestock depredation by tigers in 2009–10 from these regions [48]. If unchecked, human-wildlife conflict will escalate leading to both retaliatory killing of tigers, as well as reduced support for tiger conservation among the local communities [51], [52].

In context of the western TAL, our study highlights the need for adopting a framework that ensures the unified control of the landscape under a single conservation unit with the primary focus of managing tigers and associated wildlife. In this regard, it is important to note that recent legislations in India (Wildlife Protection Act as amended in 2006 and the Scheduled Tribes and Other Traditional Forest Dwellers (Recognition of Forest Rights) Act, 2006) reiterate the need for a combination of approaches that includes the designation of “core or critical tiger habitats” which are to be kept “inviolate” to ensure breeding of tigers, and also areas of “co-existence” with humans in the larger landscape to ensure population persistence. Therefore, integrating spatial distribution of socio-economic dependencies of local communities with the tiger occupancy model constitutes the logical next step to facilitate science-based designation of areas within the landscape into the two categories and prioritize conservation actions.

In conclusion, we demonstrate an application of the Hines et al. [20] occupancy model as a practical and robust approach to evaluate tiger occupancy across 6,979 km^2^ of potential habitat. By assessing the determinants of occupancy at the landscape scale, we have been able to identify opportunities to secure (e.g. THB II) and increase (e.g. THB I) tiger populations which assumes significance as tiger range countries embark on an ambitious plan to recover and double the range-wide population of tigers by 2022 [12].

## Supporting Information

Figure S1 **Spatial variation in wild prey index (WildP) in the western Terai Arc Landscape, India, 2009–10.**
(TIF)

Figure S2 **Spatial variation in principal prey index (PrincipP) in the western Terai Arc Landscape, India, 2009–10.**
(TIF)

Figure S3 **Spatial variation in disturbance index (Dist) in the western Terai Arc Landscape, India, 2009–10.**
(TIF)

Text S1 **Estimating the population density of tigers.**
(DOCX)

Text S2 **Simulation study to evaluate sampling site adequacy under the Hines et al. (2010) model.**
(DOCX)
