# Supplementary figures and images for: Influence of Connectivity, Wild Prey and Disturbance on Occupancy of Tigers in the Human-Dominated Western Terai Arc Landscape
Source: PLoS One. 2012 Jul 5;7(7):e40105. doi: 10.1371/journal.pone.0040105 (PMC3390357; doi:10.1371/journal.pone.0040105)

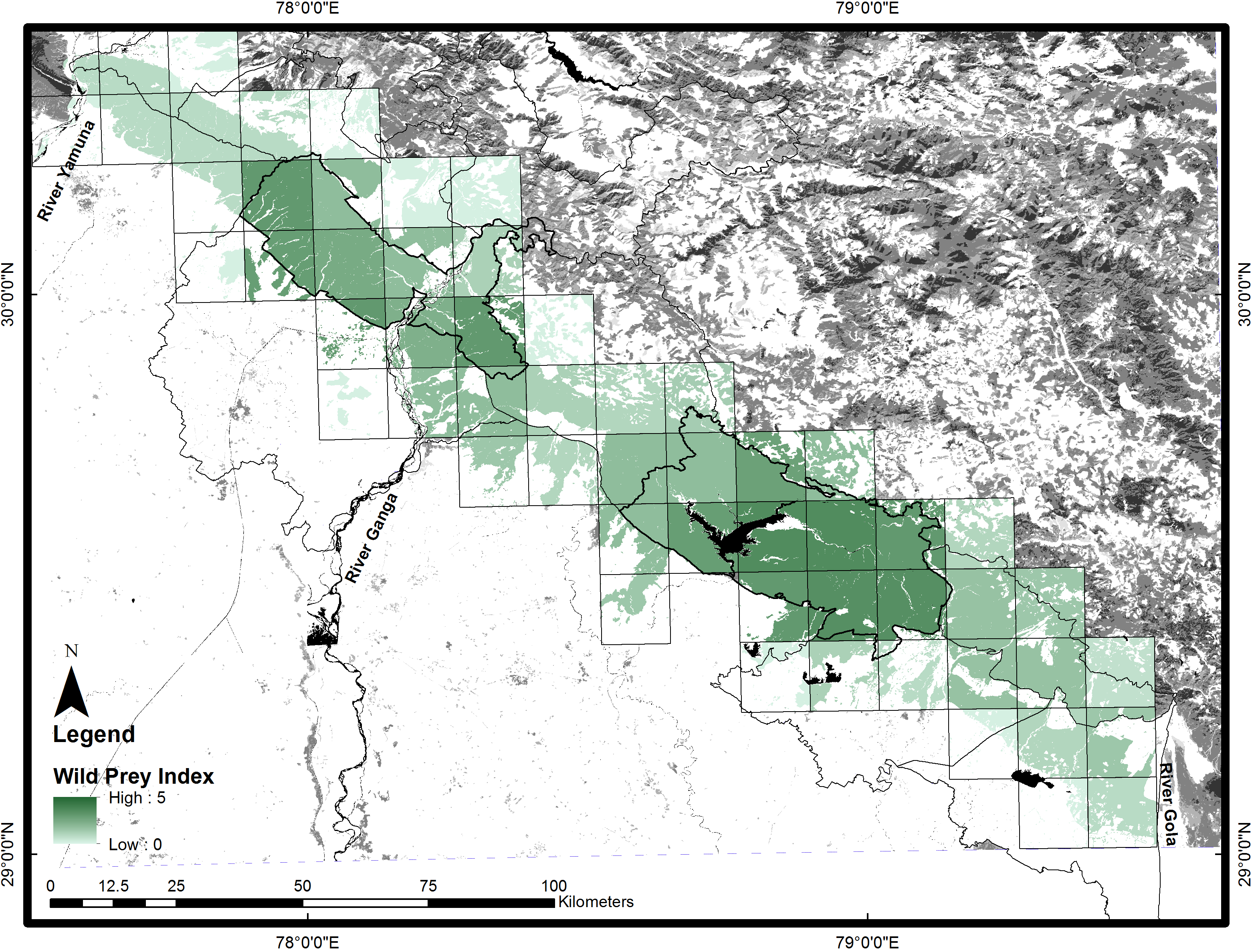

Supplement: Figure S1 — Spatial variation in wild prey index (WildP) in the western Terai Arc Landscape, India, 2009–10. (TIF) [file pone.0040105.s001.tif]

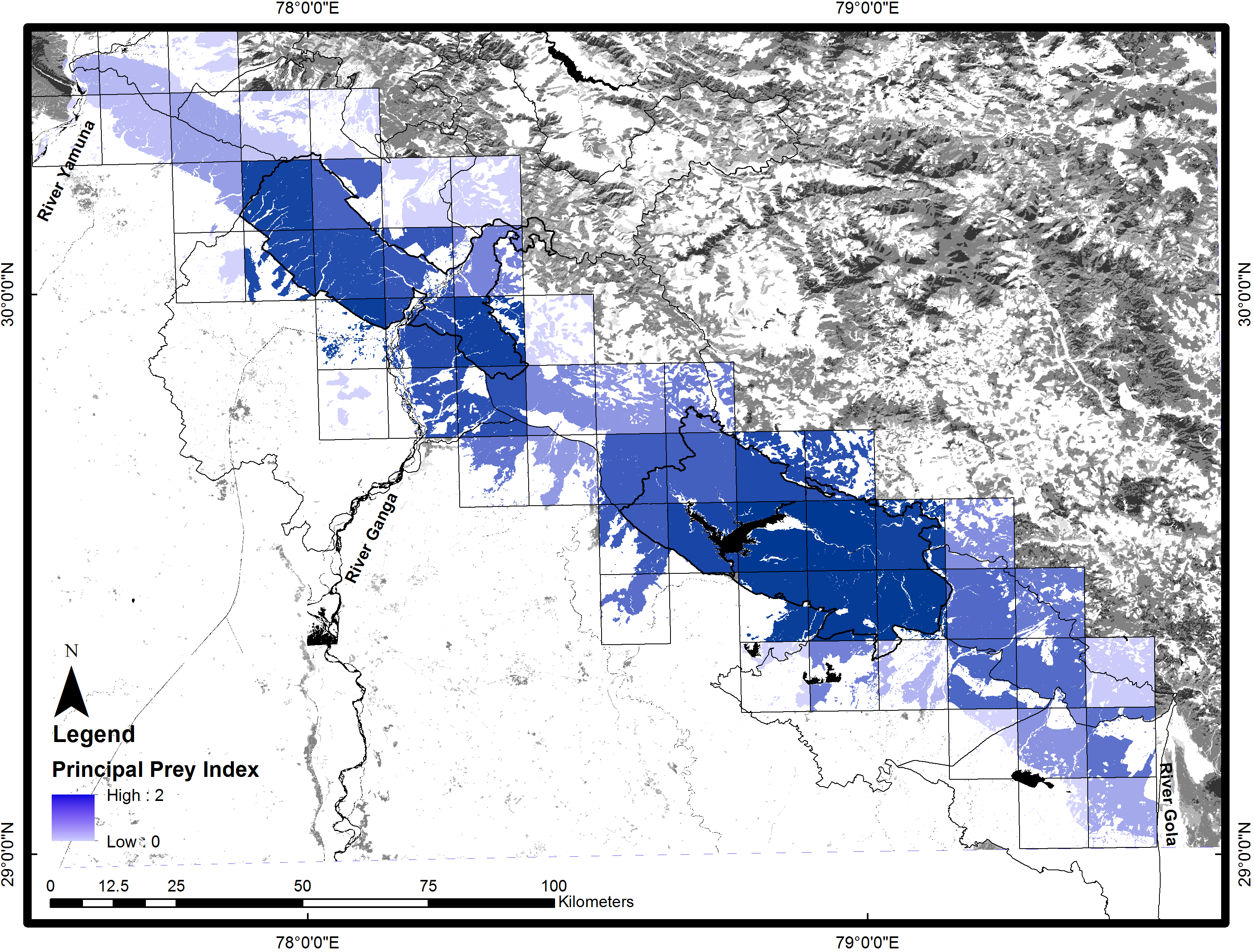

Supplement: Figure S2 — Spatial variation in principal prey index (PrincipP) in the western Terai Arc Landscape, India, 2009–10. (TIF) [file pone.0040105.s002.tif]

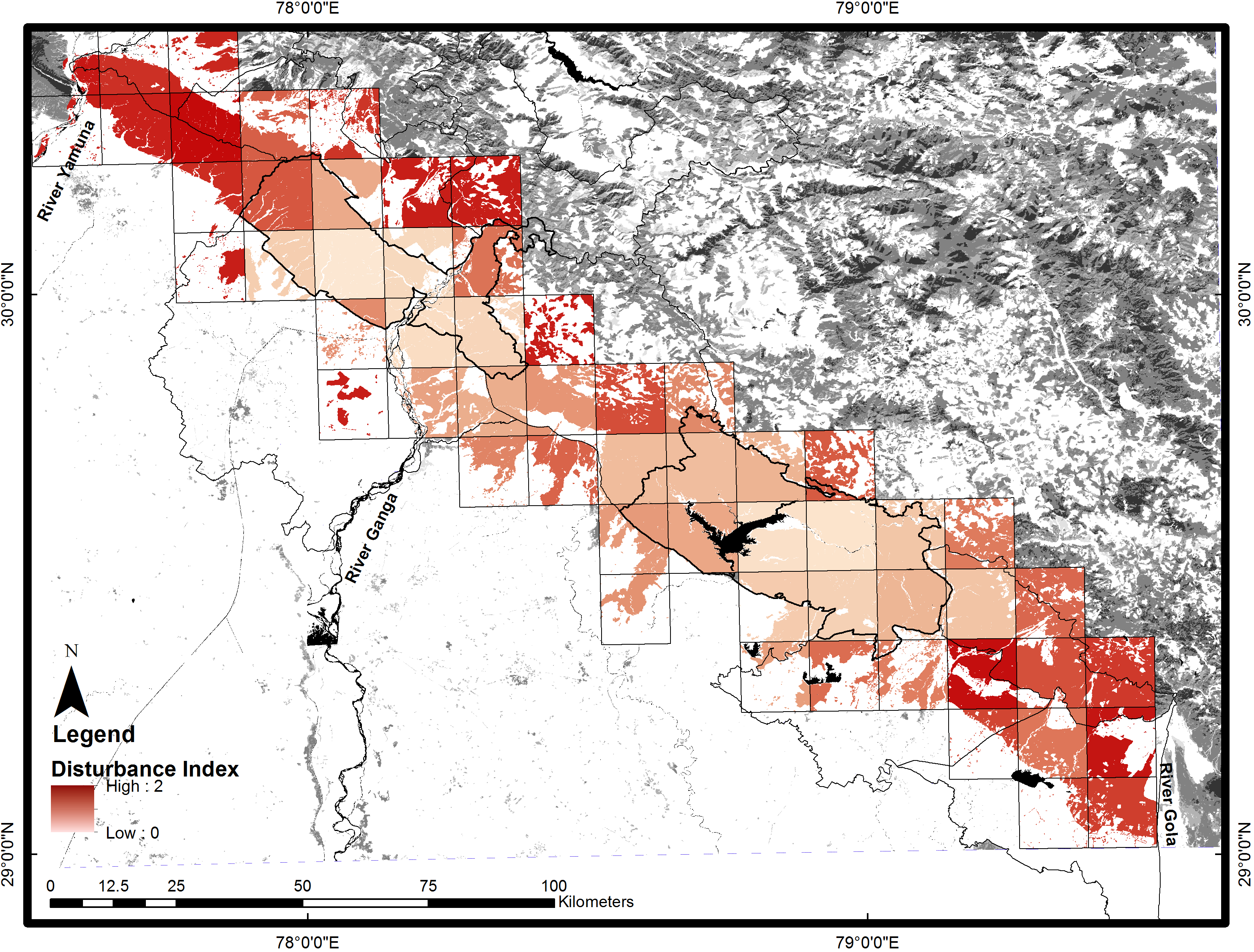

Supplement: Figure S3 — Spatial variation in disturbance index (Dist) in the western Terai Arc Landscape, India, 2009–10. (TIF) [file pone.0040105.s003.tif]
