# Supplementary material for: Influence of Connectivity, Wild Prey and Disturbance on Occupancy of Tigers in the Human-Dominated Western Terai Arc Landscape
Source: PLoS One. 2012 Jul 5;7(7):e40105. doi: 10.1371/journal.pone.0040105 (PMC3390357; doi:10.1371/journal.pone.0040105)
Supplement: Text S1 — Estimating the population density of tigers. (DOCX) [file pone.0040105.s004.docx]

**Appendix S1**

Estimating the population density of tigers

In this study we estimated tiger densities across three sites (Western RNP, Eastern RNP and Lansdowne Forest Division) in the western TAL, using photographic capture-recaptures [1]. In Western RNP, sampling was carried out using 20 trap locations across 30 days (16^th^ March to 15^th^ April 2009) within the Dholkhand east, Dholkhand west, Kansrau and Motichur forest ranges. Two sampling blocks, consisting of 10 trap sites was run for 15 consecutive days each. Prior information suggested that tiger numbers in western RNP were low and therefore we selected camera-trap locations based on previous experience [2] and other suitable sites with the presence of relatively fresh secondary evidences that indicated the use of the area by tigers. In Eastern RNP, a long term monitoring program documenting changes in tiger densities following the minimisation of anthropogenic pressures in the Chilla forest range has been on-going since the winter of 2004-05 [3,4]. For the purpose of this study we estimated densities from 2008-09 dataset. To systematically sample the area, we sampled in three blocks (spatially separated), each consisting of 10 trap sites run for 15 consecutive days. In total we sampled for 45 days during the winter (25^th^ December 2008 to 12^th^ February 2009). Finally we carried out sampling within the Kotdi and Dugadda forest ranges of Lansdowne forest division adjoining the Corbett Tiger Reserve from 13^th^ February to 31^st^ March 2010. Using a total 24 trap locations, we identified three sampling blocks each consisting of 8 trap sites run for 15 consecutive days.

Every tiger captured was given a unique identification number (e.g., RT-002) after examining the stripe pattern on the flanks, limbs, and forequarters [2,3] and the resultant encounter data was analysed using Spatially Explicit Capture-Recapture (SECR) models [5] with a 10km buffer around our trap array. We implemented this Bayesian analysis using programs R [6] and WinBUGS [7]. We employed data augmentation with 100 ‘all zero’ encounter histories and with 10,000 iterations of Markov Chain Monte Carlo (MCMC) algorithm. The posterior parameter estimates were generated from 8,000 iterations after discarding the first 2,000. In addition we estimated population size using the most appropriate model in program CAPTURE [8,9] and although Bayesian inferences of SECR models are robust compared to likelihood-based procedure, especially for small sample sizes [5], we estimated closed population density using maximum likelihood approaches (with a Half normal detection model) in the program Density 4 [10]. We do this so as to provide comparable estimates of density to those published recently by Jhala et al. [11]. The parameter estimates are presented in Table 1.

**Table 1.** Summary of population parameters estimated from camera-trapping in the western TAL.

|  |  | THB I |  | THB II | |
| --- | --- | --- | --- | --- | --- |
|  |  | Western RNP |  | Eastern RNP | Lansdowne FD |
| Number of unique individuals identified | | 2 |  | 7 | 9 |
| Total number of captures | | 6 |  | 17 | 19 |
| Estimated population size (program CAPTURE)  $\hat{N}$ (SE) | | 2 (0.3) |  | 9 (0.9) | 10 (1.6) |
| Effective trap area (km^2^) | | 266 |  | 133 | 101 |
| SECR Bayesian inference | |  |  |  |  |
|  | $\hat{N}$ (SD) | 7.1 (2.6) |  | 38.1 (13.2) | 60.2 (22.2) |
|  | $\hat{D}$ (SD) | 1.1 (0.7) |  | 5.5 (2.2) | 6.2 (2.1) |
| SECR Likelihood inference | |  |  |  |  |
|  | $\hat{D}$ (SE) | 0.4 (0.1) |  | 5.6 (1.6) | 6.1 (2.3) |

**References**

1. Karanth KU, Nichols JD (1998) Estimation of tiger densities in India using photographic captures and recaptures. Ecology 79: 2852–2862.

2. Harihar A, Prasad DL, Ri C, Pandav B, Goyal SP (2009) Losing ground: tigers *Panthera tigris* in the north-western Shivalik landscape of India. Oryx 43: 35–43.

3. Harihar A, Pandav B, Goyal SP (2009) Responses of tiger (*Panthera tigris*) and their prey to removal of anthropogenic influences in Rajaji National Park, India. European Journal of Wildlife Research 55: 97–105. doi:10.1007/s10344-008-0219-2.

4. Harihar A, Pandav B, Goyal SP (2011) Responses of leopard *Panthera pardus* to the recovery of a tiger *Panthera tigris* population. Journal of Applied Ecology 48: 806–814. doi:10.1111/j.1365-2664.2011.01981.x.

5. Royle JA, Karanth KU, Gopalaswamy AM, Kumar NS (2009) Bayesian inference in camera trapping studies for a class of spatial capture-recapture models. Ecology 90: 3233–3244.

6. R Development Core Team (2009) R: A Language and Environment for Statistical Computing.

7. Gilks W, Thomas A, Spiegelhalter D (1994) A language and program for complex Bayesian modelling. The Statistician 43: 169–177.

8. Otis DL, Burnham KP, White GC, Anderson DR (1978) Statistical inference from capture data of closed populations. Wildlife Monograph 2: 1–13.

9. Rexstad EA, Burnham KP (1991) User’s guide for interactive program CAPTURE. Fort Collins, Colorado. 29 p.

10. Efford MG, Dawson DK, Robbins CS (2004) DENSITY: software for analysing capture-recapture data from passive detector arrays. Animal Biodiversity and Conservation 27: 217–228.

11. Jhala YV, Qureshi Q, Gopal R, Sinha PR (2011) Status of the tigers, co-predators, and prey in India. Dehradun. 279 p.
