# Supplementary material for: Influence of Connectivity, Wild Prey and Disturbance on Occupancy of Tigers in the Human-Dominated Western Terai Arc Landscape
Source: PLoS One. 2012 Jul 5;7(7):e40105. doi: 10.1371/journal.pone.0040105 (PMC3390357; doi:10.1371/journal.pone.0040105)
Supplement: Text S2 — Simulation study to evaluate sampling site adequacy under the Hines et al. (2010) model. (DOCX) [file pone.0040105.s005.docx]

**Appendix S2**

Simulation study to evaluate sampling site adequacy under the Hines et al. (2010) model

In the western TAL the potential habitat for tigers in total was 6,979 km^2^. As our goal was to estimate the proportion of area occupied using Hines et al. [1] model, rather than the intensity of habitat use by tiger. We therefore chose a cell size (166km^2^) larger than the maximum home range size of ~60km^2^ [2] with the boundaries of cells coinciding with grid lines on the 1:25,000 topographic maps of Survey of India to facilitate field surveys. Given the specific objectives of our study, we wished to estimate parameters of interest ($\hat{\psi}, \hat{\theta}$, $\hat{\theta’}$ and $\hat{p}$) in two habitat blocks with differing areas (THB I = 2925km^2^ and THB II = 4054km^2^) from a total of 57 cells. Typically, occupancy modelling requires moderate to large sample sizes to achieve precise estimates [3,4]. In a previous application of the Hines et al. [1] model, inferences applied to a landscape matrix of 38,350 km^2^ framed by 205 survey cells [5]. Therefore, we carried out a simulation study to investigate the effect of number of survey sites on the bias and precision of parameter estimates [6]. Since we intended to evaluate the influence of number of survey cells, we simulated data with varying number of cells for THB I (18, 20, 36, 54, 72) and THB II (25, 37, 50, 75, 100) assuming the proportion of habitat to remain constant. In our final models, we estimated the parameters of interest from a total of 20 cells in THB I and 37 cells in THB II.

In order to test for sampling site adequacy, we simulated data in GENPRES [6] under the “single-season-spatial-correlation” model-type using the *p*(.) model variant. Keeping in mind the potential outcome of our surveys, we simulated the data to reflect the potential sampling strategy. We generated naïve estimates of occupancy and detection probability specific to each habitat block (THB I & II) using prior data [7,8] to inform our simulations (Table 1). One thousand replicates of the simulation were conducted for each habitat block and sample site, and the resulting estimates of $\hat{\psi}$ and $\hat{p}$ were recorded.

**Table 1.** Parameter values set as truth for the simulations.

THB I THB II

___________________________________ ___________________________________

Occupancy (*ψ*) Detection probability (*p*) Occupancy (*ψ*) Detection probability (*p*)

0.1 0.2 0.6 0.65

0.3 0.8

0.5 0.95

0.3 0.2 0.75 0.65

0.3 0.8

0.5 0.95

0.5 0.2 0.9 0.65

0.3 0.8

0.5 0.95

**Note:** In all simulations $\theta$ and $\theta’$ were maintained constant at 0.2 and 0.7, respectively, these estimates were derived from Karanth et al. [5].

The simulation results (Fig. 1 & 2) indicated that the estimates of occupancy were relatively unbiased (attaining a maximum value of 4.5% for *Ψ*=0.1, *p* = 0.2; n=18) and precision increased with the increase in the number of sampling sites (Fig. 1b & 2b). In general, percent relative bias for all simulation scenarios mirroring THB I (i.e. n=20) was 0.189 (SD; 0.025) and for scenarios reflecting THB II (i.e. n=37) was 0.07 (SD; 0.024). Detection probability was biased (attaining a maximum value of 7% for *Ψ*=0.1, *p* = 0.2; n=18) and estimates were relatively imprecise for all simulation scenarios mirroring THB I (Fig. 1d). Bias was relatively low for scenarios simulated with high *p* (0.65, 0.8. 0.95) and precision increased with an increase in number of sampling sites. Segment-level occupancy parameters ($\theta$ and $\theta’$) were severely biased and imprecise in scenarios with low occupancy (*Ψ*=0.1, 0.3, 0.5) and low probability of detection (*p*=0.2, 0.3, 0.5). However for simulation scenarios mimicking THB II, $\theta$ and $\theta’$ were biased low and precision improved with increasing number of sampling units. Overall our simulation results indicated that bias in occupancy for the final models would likely be trivial (% bias < |4.5%|) if the number of primary sampled sites was at least 18. Thereby indicating that our estimates derived from a total of 57 cells would be relatively unbiased.


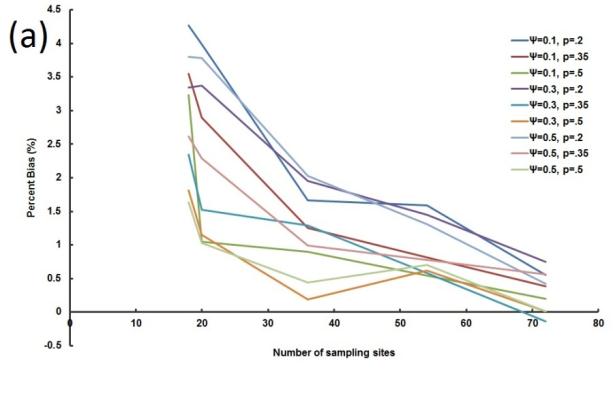

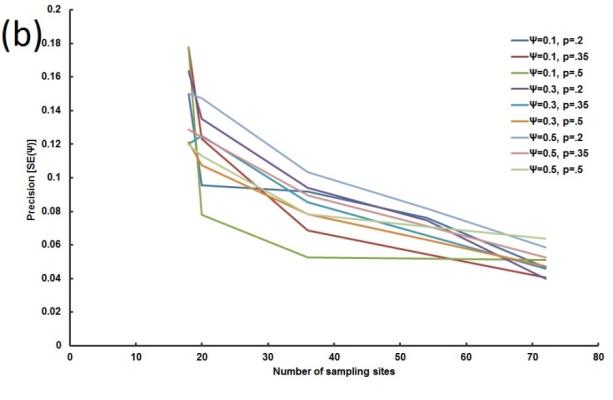


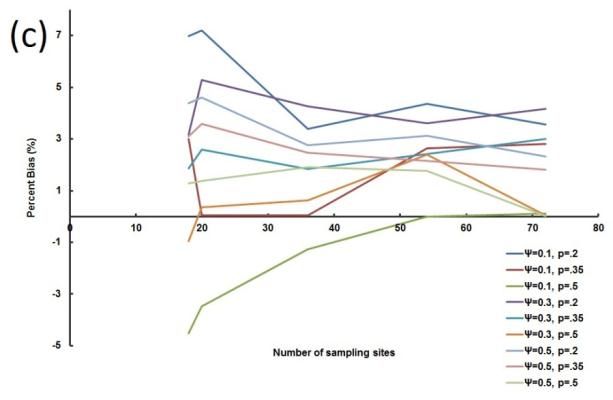

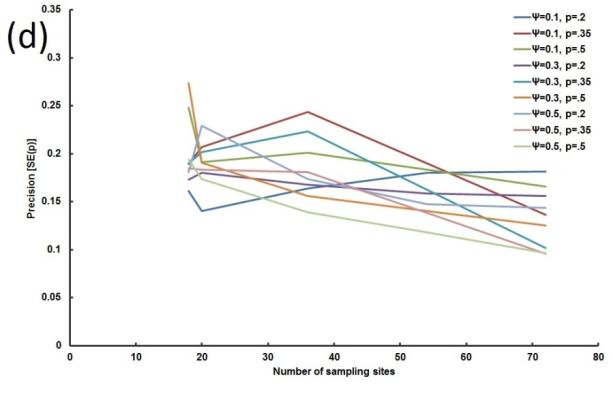


**Figure 1.** Simulation results of percent bias in estimates of probability of occupancy (*Ψ* ; a), with average estimated standard errors (SE; b) and percent bias in estimates of detection probability (*p* ; c), and average estimated standard errors (SE; d) across varying number of sites simulated for THB I.


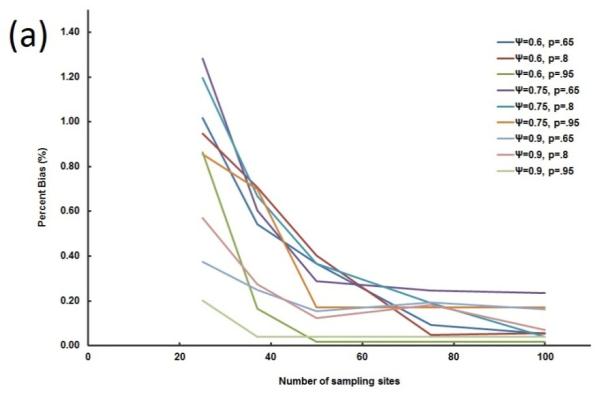

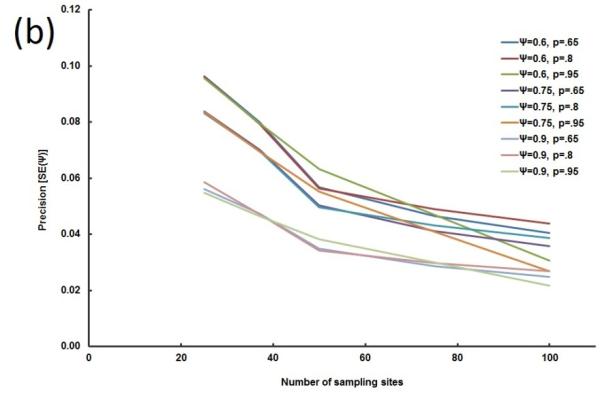


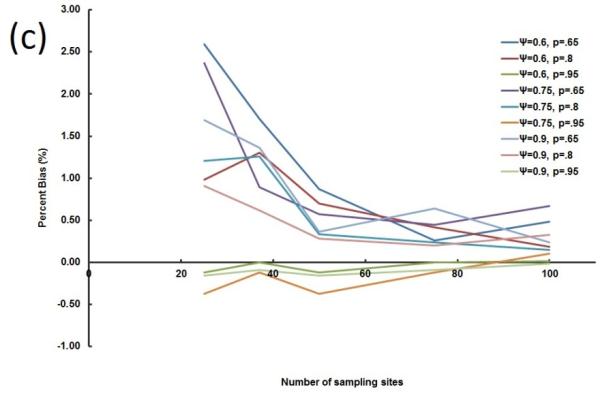

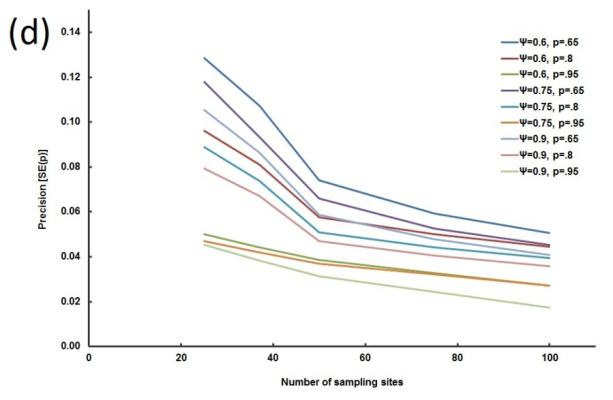


**Figure 2.** Simulation results of percent bias in estimates of probability of occupancy (*Ψ* ; a), with average estimated standard errors (SE; b) and percent bias in estimates of detection probability (*p* ; c), and average estimated standard errors (SE; d) across varying number of sites simulated for THB II.

**References**

1. Hines JE, Nichols JD, Royle JA, MacKenzie DI, Gopalaswamy AM, et al. (2010) Tigers on trails: occupancy modeling for cluster sampling. Ecological Applications 20: 1456–1466. doi:10.1890/09-0321.1.

2. Smith JLD, McDougal C, Ahearn S. (1999) Metapopulation structure of tigers in Nepal. In: Seidensticker J, Christie S, Jackson P, editors. Riding the Tiger: Tiger conservation in human dominated landscapes. Cambridge, UK: Cambridge University Press. pp. 178–189.

3. MacKenzie DI, Nichols JD, Lachman GB, Droege S, Andrew Royle J, et al. (2002) Estimating site occupancy rates when detection probabilities are less than one. Ecology 83: 2248–2255. doi:10.1890/0012-9658(2002)083[2248:ESORWD]2.0.CO;2.

4. MacKenzie DI, Nichols JD, Royle JA, Pollock KH, Bailey LL, et al. (2006) Occupancy estimation and modeling: inferring patterns and dynamics of species occurrence. Academic Press. 324 p.

5. Karanth K, Gopalaswamy A, Kumar NS, Vaidyanathan S, Nichols JD, et al. (2011) Monitoring carnivore populations at the landscape scale: occupancy modelling of tigers from sign surveys. Journal of Applied Ecology 48: 1048–1056.

6. Bailey LL, Hines JE, Nichols JD, MacKenzie DI (2007) Sampling design trade-offs in occupancy studies with imperfect detection: examples and software. Ecological Applications 17: 281–290. Available:http://www.ncbi.nlm.nih.gov/pubmed/17479851.

7. Johnsingh A, Ramesh K, Qureshi Q, David A, Goyal S, et al. (2004) Conservation status of tiger and associated species in the Terai Arc Landscape, India. Dehradun. 110 p.

8. Harihar A, Prasad DL, Ri C, Pandav B, Goyal SP (2009) Losing ground: tigers *Panthera tigris* in the north-western Shivalik landscape of India. Oryx 43: 35–43.
